# Citrus Stubborn Disease: Current Insights on an Enigmatic Problem Prevailing in Citrus Orchards

**DOI:** 10.3390/microorganisms10010183

**Published:** 2022-01-14

**Authors:** Tourya Sagouti, Zineb Belabess, Naima Rhallabi, Essaid Ait Barka, Abdessalem Tahiri, Rachid Lahlali

**Affiliations:** 1 Laboratoire de Virologie, Microbiologie et Qualité/Ecotoxicologie et Biodiversité, Faculté des Sciences et Techniques de Mohammedia, Mohammedia 20650, Morocco; sagoutitourya@gmail.com (T.S.); rallabina@yahoo.fr (N.R.); 2 Plant Protection Laboratory, Regional Center of Agricultural Research of Oujda, National Institute of Agricultural Research, Avenue Mohamed VI, BP428 Oujda, Oujda 60000, Morocco; zineb.belabess@inra.ma; 3 Unité de Recherche Résistance Induite et Bio-Protection des Plantes-EA 4707, Université de Reims Champagne-Ardenne, 51100 Reims, France; 4 Phytopathology Unit, Department of Plant Protection, Ecole Nationale d’Agriculture de Meknès, Meknes 50001, Morocco; atahiri@enameknes.ac.ma

**Keywords:** *Spiroplasma citri*, citrus, mollicutes, transmission, diagnostic, leafhoppers, Morocco

## Abstract

Citrus stubborn was initially observed in California in 1915 and was later proven as a graft-transmissible disease in 1942. In the field, diseased citrus trees have compressed and stunted appearances, and yield poor-quality fruits with little market value. The disease is caused by *Spiroplasma citri*, a phloem-restricted pathogenic mollicute, which belongs to the *Spiroplasmataceae* family (Mollicutes). *S. citri* has the largest genome of any Mollicutes investigated, with a genome size of roughly 1780 Kbp. It is a helical, motile mollicute that lacks a cell wall and peptidoglycan. Several quick and sensitive molecular-based and immuno-enzymatic pathogen detection technologies are available. Infected weeds are the primary source of transmission to citrus, with only a minor percentage of transmission from infected citrus to citrus. Several phloem-feeding leafhopper species (*Cicadellidae*, *Hemiptera*) support the natural spread of *S. citri* in a persistent, propagative manner. *S. citri*-free buds are used in new orchard plantings and bud certification, and indexing initiatives have been launched. Further, a quarantine system for newly introduced types has been implemented to limit citrus stubborn disease (CSD). The present state of knowledge about CSD around the world is summarized in this overview, where recent advances in *S. citri* detection, characterization, control and eradication were highlighted to prevent or limit disease spread through the adoption of best practices.

## 1. Introduction

Stubborn is a worldwide citrus disease that reduces the productivity and growth of affected trees [1]. Although the disease does not kill citrus trees in most cases [2,3], it has a significant economic impact [2], particularly when it infects them early in their growth cycle (severe plant stunting is observed) [2,3]. Citrus stubborn disease (CSD) was originally discovered in California Navel sweet orange (*Citrus sinensis* (L.) Osb.) trees in 1915 [4]. It was not until 1942 when stubborn was identified as a virus-like infection. Furthermore, it was only afterward that the exact nature of this bacterial pathogen was revealed [3]. The term “stubborn” refers to the reactions of buds that do not grow as expected after top-dressing sick trees. The disease was also called “acorn disease” for the numerous acorn-shaped fruits produced by infected trees [1]. The disease is present in the majority of nations where citrus grows in dry or semi-arid environments. It can be found in the warmer parts of Arizona and California, as well as in the bulk of North African, Near-Middle Eastern, and Arabian Peninsula countries. However, CSD is a rare phenomenon in cooler climates, since the vector, as well as the causative agent, are favored by warmer temperatures [1,2].

Given the importance of citrus pathology and the significant research progress in recent years, this review focuses on advancements linked to stubborn by highlighting the current knowledge on the characterization of the causal agent and the symptoms it causes, the development of reliable and speedy diagnosis methods, potential vectors, and management options, among other topics. A brief overview of the current state of CSD in the Mediterranean Basin is included, with a focus on its spread in Morocco’s citrus-growing regions.

## 2. Taxonomy, Genome Structure, and Organization

*Spiroplasma citri*, a fastidious wall-less bacterium limited to phloem, is the causal agent of CSD. The shape and motility of this pathogen are helical [5]. *S. citri* belongs to the domain *Bacteria*, phylum *Firmicutes*, class *Mollicutes*, order *Entomoplasmatales*, and family *Spiroplasmataceae* [6]. *S. citri* is a Gram-positive bacterium belonging to a phylogenetic group of microorganisms with low G-C concentration [7]. The earliest *Spiroplasma* to be isolated in pure culture, and, as a result, the first to be assigned to the genus as *S. citri*, was the causal agent of CSD [5,8]. Serological traits such as cross-serological growth inhibition and organism deformation can be used to classify members of the *Spiroplasma* group [9]. *S. citri* is classified as a member of Serogroup I, Subgroup I-1 [9,10]. With a genome size of roughly 1.8 Mbp and a single 16S-23S-5S ribosomal RNA (rRNA) operon, *S. citri* possesses one of the largest genomes among Mollicutes [11]. The total genome size of five strains of *S. citri* described by Yokomy et al. [12] ranged from 1,611,714 to 1,832,173 bp in plants and 1,968,976 to 2,155,613 bp in leafhoppers [12]. In addition, between 1908 and 2556 coding sequences were predicted in this study. In strains from the United States, one set of rRNA genes and 32 transfer RNA (tRNA) genes were predicted. This result is in line with the R8-A2 T strain, a Moroccan strain that was originally isolated from an *S. citri*-diseased sweet orange tree [13,14]. 

In addition to the circular chromosome, *S. citri* has plasmids and virus genomes that contribute to genetic information [15,16]. *S. citri*’s genome is characterized by a high adenosine-thymidine concentration and the use of UGA to encode tryptophan rather than serving as a stop codon [15,16]. The number of plasmids from plant hosts varies between one and seven [13,14,17], and eight or nine plasmids from beet leafhoppers [17]. Most plasmids were found in beet leafhopper strains, followed by carrot, Chinese cabbage, horseradish, and citrus strains, respectively. One plasmid with high similarity to plasmid pSci6 was found in all *S. citri* strains [13]. pSciA and pSci1 to pSci6 are plasmids that are replicated 10 to 14 times in each cell. Plasmids pSci1 to pSci5 encode surface proteins of the *S. citri* adhesion-related protein (ScARP) family, with pSci6 conferring insect transmissibility [18]. SpV1 was the first virus bacteriophage to infect *S. citri* and introduce DNA through horizontal transference. The biological significance of viral sequences introduced into cells is currently unknown. In contrast, the physical map of the *S. citri* genome indicates that this bacteriophage might be present in up to 17 copies within the genome, representing up to 8% of the total genome content [19,20]. The *S. citri* chromosome can be entirely or partially integrated by this single-stranded circular DNA virus [19]. SpVl-like sequences are involved in large-scale genomic rearrangements such as inversions, transpositions, and deletions of vast DNA regions, as well as chromosome size changes [21]. The presence of prophage sequences in the genomes of *S. citri* could help it broaden its host range [13].

DNA acquisition and loss, DNA replication and repair, homologous recombination, and transposition are all factors in the genetic diversity of *S. citri* [16]. Due to chromosomal and extrachromosomal inversions and deletions, graft transfer or many passages in medium cultures can lead to genome changes [22,23]. For example, due to chromosomal inversion and genomic deletions in the BR3-3X strain of *S. citri*, continual graft transmission from periwinkle (*Catharanthus roseus* (L.) G. Don) to periwinkle resulted in a lack of transmissibility by the natural vector leafhopper *Circulifer tenellus* Baker (synonym: *Neoaliturus tenellus* Baker). The high passage in the artificial medium also affects the transmissibility of *S. citri* [22,24]. Crucially, transcriptional gene regulation in *S. citri* likely plays a key part in its ability to adapt to its hosts and could be a useful tool for altering the spiroplasma surface in response to changing environmental conditions [25].

The genome of *S. citri* GII-3 (1820 Kbp) [18], a strain obtained from its Moroccan leafhopper vector *Circulifer haematoceps* Mulsant and Rey (synonym: *Neoaliturus haematoceps* Baker) [26], encodes 645 membrane proteins, including 68 putative lipoproteins [27], and 577 transmembrane proteins [28]. Many mycoplasma species’ interactions with their hosts have been discovered to be dependent on membrane proteins, particularly surface proteins [29,30]. Spiralin is an amphiphilic polypeptide with an apparent molecular size of 26 to 28 KDa that is the most abundant protein in the *S. citri* membrane [31,32]. Spiralin is not required for disease or motility, but it is requested for successful spiroplasma transmission via the insect vector [33,34]. The central coding region of the 9.6 Kbp sequence of *S. citri* BR3-3X—of which one copy was suppressed in the insect’s non-transmissible lineage, BR3-G—contained several genes, including the putative membrane protein P58 coding gene [35]. This gene contained its proper promoter and terminator signal sequences for transcription and the Shine–Dalgarno sequence for translation. Spiroplasma-insect vector interactions are mediated by the P58 protein. However, the lack of a copy of the P58 gene in the insect-non-transmissible mutant line, BR3-G, might not explain the resulting loss of its transmittance to insects. The non-deleted copy of this gene in BR3-G appears to be functional, as the P58 protein was detected in this line [15]. Extrachromosomal DNA with a high molecular mass carries the P32 gene, which corresponds to a bigger plasmid of 35.5 Kbp. The P32 gene may have a role in the transmission and might be used as a marker for the transmissibility of *S. citri* by leafhoppers [36]. In spiroplasma, there was a link between the loss of high-molecular-mass plasmids and the non-transmissible phenotype [18]. P89 (Sarp1), a potential adhesion-related protein from *S. citri*, is found on a plasmid and in the pathogen genome [37,38,39] and is involved in *S. citri*’s adhesion to vector cells, specifically *C. tenellus* cells [38]. The surface lipoprotein Sc76, homolog to a solute-binding protein of an ABC transporter, was also identified to be relevant, as disrupting the gene drastically reduced *S. citri*’s ability to be transmitted by *C. haematoceps* [40]. There are 466 amino acids in the Sc76 gene product (51.8 KDa). This gene is involved in the transport of glucose [41].

Molecular phylogenetic inference of 39 spiroplasmas was performed utilizing the NCBI database’s 16S rRNA genes. The *S. citri* strains are closely related, but not identical, according to this gene sequence analysis. *S. citri* strains formed a monophyletic group with plant pathogenic *Spiroplasma kunkelii*, *Spiroplasma phoeniceum*, and a honeybee pathogen, *Spiroplasma melliferum*, according to the 16S rRNA gene phylogeny [13]. Citrus strains C189 from southern California and R8-A2 from Morocco were clustered together in phylogenetic analyses using core orthologous sequences among *S. citri* strains. CC-2, a Chinese cabbage isolate, and C5, a carrot isolate, belong to the same group. The strains LB 319 (citrus), BLH-13 (beet leafhopper), BLH-MB (beet leafhopper), and BR12 (horseradish) formed a distinct clade [13].

## 3. Symptoms and Economical Impact

**Figure 1:**
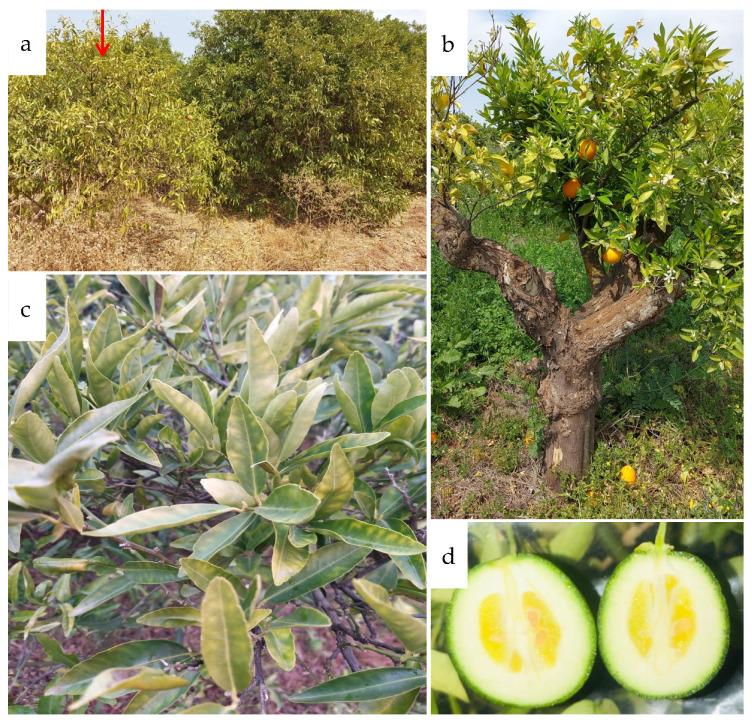
Field symptoms of citrus stubborn disease observed in Moroccan citrus orchards in the Tadla region (situated in the center of Morocco) in growing season 2021. (**a**) Compressed and stunted Navel sweet orange tree (red arrow); (**b**) clementine tree with different phenological stages; (**c**) Navel sweet orange leaves showing nutritional deficiency-like symptoms; (**d**) Navel sweet orange fruits with a gland shape.

CSD symptoms are similar to those caused by other biotic and abiotic stresses, thereby making it difficult to distinguish between a diseased and a healthy *S. citri* tree [42]. In most cases, stubborn-infected trees do not die, but instead, establish a state of equilibrium. The growth of the trees slows and the tops of the trees flatten. Flowering can occur at any time of year, but the fruits are of poor quality, and their number declines over time [43]. The fruits are non-homogeneous in color, have a gland shape, and a green stylar end (Figure 1) [44,45,46]. Numerous papers have described the symptoms reported to be associated with CSD [2,3,14,43,45,47,48,49,50,51,52,53,54]. Multiple axillary buds, a large number of shoots, and erect, bunchy growth with short internodes are all signs of aberrant growth. On severely damaged trees, twigs become stunted and die, and they become more susceptible to both cold and heat. While lower branches of some diseased trees show symptoms, many trees can still provide typical-looking leaves and fruits on their shaded lower limbs. In some cases, trees show symptoms of CSD only on one or a few branches and may remain in this state for years. Infected trees have smaller leaves than healthy trees. The leaves might have a healthy or diseased appearance (mottled, cup-shaped, distorted in different ways, and pinched in near the tip). It is worth noting that diseased trees’ mottled leaves look a lot like leaves with zinc, iron, or manganese deficiencies (Figure 1).

In severely damaged branches, premature leaf drop can occur. Out of season, diseased trees may have several phases of limited blooming, resulting in the appearance of fruits at various stages of maturity. The produced fruits may be of a smaller size with an asymmetric shape (acorn shape), display greening at the stylar-end, and have a high level of seed abortion, notably in Valencia oranges. Two types of symptoms have been identified, depending on the severity of the condition: (*i*) “severe”, when the entire tree canopy is damaged, i.e., all branches have mottling and short internodes, and many show off-season blooming; and (*ii*) “mild”, when the tree is practically asymptomatic or the symptoms, such as short internodes and leaf mottling, are limited to a few branches [42,52].

All these symptoms are likely related to the fact that *S. citri* needs energy supplies (such as sterols and carbohydrates) from its host plant to grow [55,56]. *S. citri* competes with its host for these energy sources while living in the plant, leading to the depletion of some hormones and sugars and the accumulation of others [52]. Furthermore, it has been demonstrated that *S. citri* mutants that are unable to utilize fructose have been shown to exhibit only modest and delayed symptoms. This is because fructose uptake by sieve-tube-restricted wild-type spiroplasmas is believed to deplete companion cells of fructose, thus suppressing sucrose loading in sieve tubes [41]. As a result of this imbalance, the normal citrus plant metabolism is disrupted, resulting in CSD symptoms (stunting, leaf mottling, reduction in fruit size and number, and the occurrence of off-season blooming) [52].

Numerous elements, particularly environmental conditions, may alter the symptom expression of CSD [57]. It is critical to note that elevated temperatures enhance the symptoms of CSD [2,58,59]. In other words, warm temperatures (27 °C at night and 35 °C during the day) cause the mottled-leaf symptom of CSD on sweet orange and grapefruit to appear within 2 months, whereas cool temperatures (23 °C at night and 27 °C during the day) tend to delay the emergence of the same symptom (up to 5 months) [58]. Bové et al. [59] observed similar results on Madam Vinous seedlings. Warm temperatures (27 °C for 8 h nights and 32 °C for 16 h days) have been found to promote the onset of severe CSD symptoms on Madam Vinous seedlings within 5 weeks, whereas cool temperatures (22 °C for 8 h nights and 24 °C for 16 h days) tend to induce only mild symptoms after a long period (26 weeks). It is worth noting that the classic symptoms of CSD, such as tiny, cupped leaves with pale-green tips and mottling, were only seen in warm weather [59]. Calavan and Bové [60] suggested that symptom severity was linked to bacterial titer and/or strain virulence. Further, Mello et al. [61] found that CSD severity is related to *S. citri* titer but not to bacterial genotype. Indeed, the titer of *S. citri* in fruits harvested from severely symptomatic trees is about 6000 times higher than in fruits harvested from mildly symptomatic trees. It is worth noting that the genotypes found in this study were found in trees that were both severely and mildly symptomatic. This confirms that genetic differences in *S. citri* populations have no impact on disease severity [61]. CSD may be influenced by the rootstock chosen. Indeed, a study conducted in Sicily to assess the vulnerability of four rootstocks to CSD (Cleopatra mandarin (*Citrus reshni* Tanaka), Rangpur lime, sour orange, and Volkamer lemon (*Citrus volkameriana* V.Ten. and Pasq.)) revealed that *C. volkameriana* is susceptible to CSD. The inoculated seedlings of the three other rootstocks showed no significant variations in vegetative growth or other signs on leaves and stem after one year. However, the *C. volkameriana* seedlings showed a temporary decline in growth five months after inoculation [57]. CSD severity may also change depending on the citrus cultivar. This was demonstrated in a three-year study conducted in California to monitor CSD progress in 12 orchards. In two orchards of grapefruit, the severity of CSD increased drastically from 0 (healthy) to 3 (26–50% of a tree showing symptoms), although the Valencia orange showed the smallest increase in disease severity. The CSD severity reaction in Navel orange was middling. It is worth noting that in the second year of testing, Navel and Valencia sweet orange trees showed a pronounced stubborn symptom remission for about four months [47]. 

Several field trials on various citrus species, cultivars, and rootstocks have been performed in various agroecosystems to assess the impact of CSD on vegetative growth and yield (Table 1). This is the case, for example, with 12-year-old Navel sweet orange trees that were naturally infected with *S. citri* and whose fruit yield was investigated. The yield was higher in trees with minor CSD symptoms, according to the findings. On the other hand, trees with mild symptoms yielded 20 Kg less, on average, than healthy trees [62]. The fruit yield of CSD-infected Navel and Valencia sweet orange trees was likewise significantly reduced [63]. Diseased trees of both cultivars had a considerable loss in mean fruit weight when compared to healthy trees, with reductions of about 19 and 34% for Navel and Valencia, respectively [63]. Recent research on Navel oranges has revealed the impact of CSD on fruit production [52]. The disease’s impact on Navel sweet orange production was underlined by the findings, which revealed that diseased trees, particularly those with severe symptoms, show a considerable reduction in fruit number. In other words, in 2006 and 2007, the productivity of *S. citri*-positive trees was 25% and 32% lower than that of *S. citri*-free plants, respectively. It is worth noting that the disease had a greater influence on Navel yield on severely symptomatic trees (52 and 45% lower in 2006 and 2007, respectively) than on mildly symptomatic trees (no statistical difference). On *S. citri*-infected trees, yield reduction might be attributed to both earlier fruit drop and the production of lighter and smaller fruit than on *S. citri*-free trees [52]. Furthermore, CSD has been associated with reductions in fruit size, particularly with Valencia oranges [54]. Further, the CSD has also an impact on tree height and canopy diameter [52]. 

The available studies on the impact of CSD on fruit quality and quantity are inconclusive. Indeed, Mello et al. [52] and Kyriakou et al. [63] reported that both juice quantity and quality are not affected by CSD. The *S. citri*-positive trees, on the other hand, produced insipid, sour, or bitter-tasting fruits [65]. Furthermore, CSD is regarded as one of the primary causes of citrus fruit quality degradation in Egypt [46]. Indeed, *S. citri*-positive trees produced fewer, lower-quality fruits (reduced size and asymmetric shape) than *S. citri*-negative trees [52].

## 4. Transmission and Epidemiology

The citrus stubborn spiroplasma is vectored by many leafhopper species. The disease is also propagated by grafting or collecting bud material from diseased plants. Several factors, associated with the causal agent, its plant hosts, vectors, management practices, and the environment, influence the disease epidemiology. Indeed, the transmission level of *S. citri* is correlated with temperature and is higher in warm conditions [59,66]. *S. citri* is an obligate parasite that lives in the phloem sieve tubes of infected plants [53,67]. Leafhoppers have been proven to transfer the mollicute from and to a wide range of weeds and vegetable hosts [68]. Infected weeds became stunted and yellow, and as they dried up in warm or hot temperatures, *S. citri* vectors moved from these hosts to young citrus trees, which are more susceptible than older ones. Transmission is primarily from infected weeds to citrus, with less transmission from infected citrus to citrus [69]. Infection with *S. citri* can also be spread through grafting using contaminated scions [4]. As a result, in areas where the disease is not endemic, the use of *S. citri*-free buds is required to prevent infection [70].

In Arizona and California, ornamental periwinkle seedlings were the first hosts to be naturally found infected with *S. citri* [71,72], and they were later used experimentally to learn more about the spiroplasma’s natural transmission in both Morocco and Syria [71,72,73,74,75,76]. It is vital to note that periwinkle is employed as a model host plant for mollicutes research in plant pathology. This is due to the ornamental plant’s high susceptibility to phytoplasma and spiroplasma infection from various crops [77], which can be transmitted by insect vectors feeding on *S. citri*-infected trees [78], dodder transmission [79], and/or mechanical inoculation [80]. A rapid reduction in the size and number of plant flowers, reduction in leaf size, chlorosis of leaf tips and margins, stunting, and mortality are all indicators of *S. citri*-infected periwinkles [81].

Leafhoppers belonging to the *Cicadellidae* family (*Deltocephalinae* subfamily) are responsible for persistent and propagative insect transmission of *S. citri* [82,83]. *C. haematoceps* is the main vector of *S. citri* in the Mediterranean region. Turkey, Morocco, Syria, and France (Corsica) have all reported it [76]. *C. haematoceps* is also the most common species in Asia, particularly Iran, however it appears that the beet leafhopper, *C. tenellus*, is more common there than in the Mediterranean Basin. As a result, both *C. haematoceps* and *C. tenellus* can be vectors in the Middle East. The major vector of *S. citri* in the United States is *C. tenellus* [69]. Indeed, *S. citri* was found to be transmitted to citrus and periwinkle by a beet leafhopper collected in California citrus plantations [84]. *C. haematoceps* host plants have been identified in Syria and France, stating that their presence along the Mediterranean coast explains various epidemic scenarios, including those in citrus fields with nucellar trees [69].

Crossing the insect vector’s intestinal and salivary gland barriers is required for the persistent and propagative transmission of *S. citri*. These crossings are based on an endocytosis/exocytosis mechanism wherein bacterial protein complexes identify certain patterns on eukaryotic cell surfaces [85]. The *S. citri* infects the entire insect via crossing a circulative route after being acquired from the phloem vessels of an infected plant by leafhopper vectors. Spiroplasmas enter the insect gut wall, multiply, circulate, and infiltrate most of the insect organs, including the salivary glands, before being discharged into the primary salivary duct leading to the stylet’s salivary canal. During feeding, they are delivered into the plant phloem with salivary secretions [86,87,88]. *S. citri* multiplies in the phloem sieve components of the host plant, causing severe symptoms [25]. *S. citri* was found to lose its ability to cross the gut and salivary gland barriers and to be transmitted after numerous plant grafts or several sub-cultures in in vitro culture without insect passage [24]. The absence of several proteins (146, 144, and 92 KDa) thought to be important in transmission explains this study [39]. *S. citri* transmission by insect vectors has been linked to several proteins. Spiralin [33,89], Sc76 (the solute binding protein of an ABC transporter) [40], and P89, are all encoded on pBJS-O plasmid [38,90]. In vitro, the P89 protein was found to be necessary for insect cell adhesion [91]. Further, the *S. citri* transmission also involves P58 [15] and P32 encoded on plasmid pSci6 [36,37].

## 5. Methods to Detect the Disease

### 5.1. Biological Indexing

Calavan and Christiansen [92] developed biological indexing of CSD for the first time in 1965, where sensitive varieties such as Madam Vinous or Pineapple sweet orange were inoculated with the examined tissue [92]. To ensure the effectiveness of indexing, these citrus cultivars, known as indicator plants, should be kept at warm temperatures [93]. Both side and young leaf piece grafts have been demonstrated to successfully transmit the disease to Madam Vinous sweet orange indicator plant seedlings. These two inoculum sources were shown to be more effective than buds or blind buds in disease transmission [94,95]. Madam Vinous seedlings used as indicator plants produced new growth within 3 weeks and displayed significant symptoms of CSD within 5 weeks when kept in a greenhouse under warm conditions (30 °C to 40 °C maximum days and 26 °C to 27 °C night). It is worth noting that the disease’s classic symptoms, such as small, cupped leaves with pale-green tips and mottling, were only seen in hot climates [59,96]. Mannaa et al. [46] developed an effective biological indexing technique called inverse inoculation. This approach allows the symptoms of CSD to be observed only 4 weeks after inoculation and improves transmissibility (85%) compared to standard inoculation, which takes 3 months and has a low success rate (35%) of transmission [46]. It is worth noting that biological indexing has revealed the irregular distribution of *S. citri* throughout the plants, making this technique difficult to utilize regularly. However, due to the presence of mild forms of *S. citri* on the one hand, and the uneven distribution of the causal agent on the other, this observation helps to explain why some experimentally infected plants do not develop symptoms [48,94,97,98,99,100].

The use of biological indexing in CSD diagnosis has two fundamental drawbacks. The first is *S. citri*’s low rate of greenhouse transmission. The standard approach of biological indexing on indicator host plants was used to calculate this rate. The second is the length of time it takes for the symptoms to develop on the indicator plants. The prior limits could be explained by the low concentration of *S. citri* in the bud stick used, especially during cold seasons. To address the problem, adjustments to the standard method of biological indexing must be undertaken to boost the rate of successful pathogen greenhouse transfer [46]. In addition, biological indexing, which includes mechanical inoculation, must be supplemented by laboratory indexing, including serological, molecular, and chemical assays [51].

### 5.2. Isolation and Culturing

Two research groups were the first to describe *S. citri* in in vitro culture [101,102]. Since then, *S. citri* isolation and culture have been used to diagnose CSD in field trees as the gold standard [4,103]. For initial isolation and routine cultivation of *S. citri* from both plant material and leafhopper hosts, several culture mediums have been devised. C-3G [104], LD8 [105], SP4 [106], and R2 [107] are just a few examples (Table 2). The requirement of cholesterol for growth, as well as a total resistance to penicillin, are the two most important cultural characteristics [103]. Most mycoplasma mediums contain complicated elements such as basis compounds (PPLO broth base, animal serum, and yeast extract), as well as other substances (tryptone, peptone, and animal tissue culture medium) that are frequently included. Other, simpler media have been created, such as R2 [107].

*S. citri*, like other bacteria, has a sigmoid growth pattern. At 29 °C, helix doubling takes 20 h in the exponential phase. When the pH of the culture medium dropped to 5.4 or below, *S. citri* lost motility and helicity [108]. A second culture is required after 2 days to ensure the ongoing growth of *S. citri*, with the maximal concentration of inoculum in the second cultivation is required after 2 weeks [104]. In 0.8% agar media, fried egg-shaped colonies (small in size and round in form) can be seen [44,51,104]. During the exponential phase, filament branching was common, and the helical filaments stuck to each other and formed aggregates in the old cultures [108].

**Table 2:** Summary of the major medium culture used for *Spiroplasma citri* isolation and growth.

Ingredients	Medium Name [References]				
	C-3G [104]	R2 [107]	M1D [109,110]	LD8 [105]	SP4 [106,111]
Distilled water	72 mL	76 mL	Fill to 100 mL	1.2 mL	61.5 mL
PPLO broth base w/o	1.5 g	1.5 g	-	1.2 g	0.35 g
Mycoplasma broth base (BBL)	-	-	700 mg	9 g	0.35 g
Sucrose	12 g	8 g	332 mg	6 g	-
Glucose	-	-	33.2 mg	400 mg	0.5 g
Fructose	-	-	33.2 mg	0.4 g	
Phenol red (0.2%)	1 mL	1 mL	1 mL	-	2 mL
Horse serum	20 mL	15 mL	-	-	
Fetal bovine serum (heated at 56 °C for 1 h)	-	-	16.6 mL	10 mL	17 mL
Penicillin (1 MU/g)	100 mg	100 mg	100 mg	-	100 mg
Sorbitol	-	-	2.5 g	-	-
Peptone	-	-	266 mg	-	0.53 g
1.0 n NaOH	-	-	As needed	-	-
Schneider’s insect medium	-	-	53.4 mL	-	-
Tryptone 10.0 g	-	-	332 mg	-	1 g
CMRL 1066 medium (10×) (with glutamine) (Gibco 154)	-	-	-	-	5 mL
Fresh yeast extract (25% solution)	-	-	-	5 mL	3.5 mL
Yeastolate (2% solution, sterile)	-	-	-	0.2 mL	10 mL
HEPES buffer	-	-	-	1.5 mL	-
Organic acids	-	-	-		-
α-Ketoglutaric acid				0.04 g	
Pyruvic acid				0.04 g	
Inorganic salts	-	-	-		
KCl				0.04 g	
KH_2_PO_4_				0.03 g	
MgSO_4_ 7H_2_O				0.02 g	
NaCl				0.14 g	
NaHPO_4_				0.02 g	
NaSO_3_				0.05 g	
Amino acids	-	-	-		
L-Arginine				0.06 g	
L-Asparagine				0.06 g	
L-Cysteine HCl				0.04 g	
L-Glutamine				0.06 g	
Methionine				0.04 g	

Although *S. citri* isolation and culture are a very sensitive and specific method for CSD diagnosis, its time-consuming nature (it takes 2 to 3 weeks) prevents its routine usage. Furthermore, contamination by non-target organisms is regarded as a limitation that could limit the widespread application of this diagnostic approach [112].

### 5.3. Antibodies-Antigen-Based Methods

*S. citri* was found to be detectable in infected host plant tissue, arthropod hosts, and pure culture using an enzyme-linked immunosorbent assay (ELISA) [113,114,115]. Indeed, ELISA has been the most often used diagnosis technology in the preliminary sanitary evaluation of propagating material due to its ease of use and ability to evaluate a large number of samples [116]. ELISA’s sensitivity is comparable to that of culture. In citrus, both assays can detect *S. citri* in 95% of symptomatic nursery or field trees in citrus [117]. The tests are not sensitive enough to accurately detect *S. citri* in trees that do not show any symptoms. The limited number of spiroplasmas present in the phloem makes the early detection of the pathogen difficult during the plant disease establishment [117,118]. Furthermore, when utilizing citrus leaves as samples, the results were uneven [114].

The immunocapture-based polymerase chain reaction (IC-PCR) is thought to be a promising technology for detecting low levels of *S. citri* in citrus plants and insect cells. Indeed, the sensitivity of IC-PCR has been compared to that of two other techniques, ELISA and polymerase chain reaction (PCR). The lowest number of spiroplasmas detected per milliliter (mL) of plant extract was roughly 10^−6^ to 10^−7^ per mL using ELISA. By contrast, PCR found around 10^−4^ spiroplasmas per mL, while IC-PCR detected approximately 10^−3^ per mL [119]. Two primer pairs were used to provide an enhanced IC-PCR test: D/D’ [120], which is specific for the entire spiralin gene, and SC/SC’, which is specific for a portion of the spiralin gene. The two primer pairs amplified 1,035 bp and 330 bp from infected but not healthy citrus trees, respectively [121]. In a short, IC-PCR is a sensitive and specific method for detecting *S. citri* [121].

Because CSD diagnosis is difficult due to low and variable concentrations of *S. citri* in diseased trees and the random distribution of *S. citri*, a new diagnostic approach based on an *S. citri*-secreted protein as a detection marker has been devised. This approach is based on the fact that microbial pathogens, such as *S. citri*, release a large number of proteins during infection. In diseased plants, the vascular flow allows systemic dispersion. As a result, the presence of these proteins may be more widespread than just pathogen infection sites, and they could be used as biological detection markers. With mass spectrophotometry analysis, a novel secreted protein for *S. citri* has been identified, which is strongly expressed in the presence of citrus phloem extract. ScCCPP1, an antibody raised against this protein, was able to differentiate diseased citrus and periwinkle plants from healthy ones. In summary, using the secreted protein as a marker is a successful diagnostic strategy for large-scale CSD field surveys. For this application, however, extra validation and specificity confirmation tests of the approach are required. This is because this technique can create critical data, making it unsuitable for use in the field. This can arise, for example, when field samples are taken from other citrus species, at various tree ages and stages of development, from trees infected with other bacteria and viruses associated with citrus, at various times of the year, and in various geographic locations [80]. Using the antiserum ScCCPP1, a simple direct tissue print assay has been developed. This technique was used to evaluate six adult trees that were known to be naturally infected with CSD. All of the examined plants developed positive signals from the phloem area of the stem print utilizing ScCCPP1. Using direct tissue prints on a nitrocellulose membrane, diseased *S. citri* trees were effectively diagnosed in a highly specific and reliable manner. It is worth noting that in the field, this direct print tissue assay is more sensitive than real-time PCR in diagnosing CSD. To put it another way, the results of real-time PCR did not match those of the imprint data (eight positive samples with direct tissue print assay instead of only six with real-time PCR). Positive signals have been detected without the presence of *S. citri* cells in phloem-rich tissues. This finding suggests that the pathogen detection signal was disseminated throughout the phloem via the transportation flow [80].

### 5.4. Nucleic Acid-Based Methods

CSD field diagnosis is typically challenging since culture on artificial media, biological indexing, or serological techniques to detect the causal agent are laborious, expensive, and/or time-consuming [122]. As a result, molecular assays based on PCR have been designed to address the major limitations of serological testing and culture assays for the identification of CSD (sporadic distribution of the mollicute in infected plants, seasonal concentration changes of *S. citri*, etc.) [122]. The sensitivity of PCR is 100 to 1000 times higher than with ELISA or culture assays [118,122]. Using primers amplifying the spiralin gene or multicopy genes encoding membrane proteins, several PCR-based detection techniques have been devised. P58 and P89 are among the genes used for this purpose [120,122]. Table 3 lists all of the PCR and associated assays that have been developed to detect *S. citri*. Yokomi et al. [122] found that primers based on P89 and P58 were at least 1000 times more sensitive in recognizing *S. citri* in field samples than those based on the spiralin gene [120]. *S. citri* PCR assays are highly sensitive, need a small amount of sample, and can be reliably conducted at high throughput [122]. Other primers have also been designed to target viruses associated with *S. citri* [19,112,118,122,123]. Depending on the *S. citri* isolate and the targeted gene, the detection method’s performance varies [44].

Highly sensitive and reliable real-time PCR assays were developed to address the problem of the non-detection of some *S. citri* isolates by the traditional PCR test previously developed by Yokomi et al. [122]. A real-time PCR assay based on sequences from the P58 putative adhesin multigene of *S. citri* enhances sensitivity from 8 × 10^−5^ to 1.2 × 10^−6^ ng of *S. citri* DNA (6.14 × 10^5^ to 9.6 × 10^3^ copies of target gene) per milligram (mg) of field citrus tree tissue. It is worth noting that the titer of *S. citri* was consistently higher in the fruit columella than in the leaf midribs, making the former the best choice for sampling [125]. Another primer pair (Scif/Scir) has been designed to target the pE gene of the pSci1 plasmid of *S. citri*. The real-time PCR test developed with this primer pair was more sensitive than those based on the spiralin gene (D/D’ and F1/R1) or 16S ribosomal DNA (rDNA) (ScR16F1/ScR16R1 and ScR16F1A/ScR16R2) [120,123,128]. Additionally, designing primer pairs has resulted in the development of a very sensitive and reliable real-time PCR test. This includes Php-orf1, which targets conserved prophage sequences in the *S. citri* genome [126]. It is crucial to note that this test improves the sensitivity of detection of *S. citri* by 4.91 and 3.65 cycle threshold (Cq) units, respectively, as compared to spiralin and P58 putative adhesin gene housekeeping gene primers [126].

The *S. citri* strain diversity can be studied using restriction fragment length polymorphism (RFLP) analysis of 16S rDNA, amplified by nested PCR with *S. citri* 16S rDNA-based primers [123]. The restriction enzymes AluI, HhaI, HaeIII, and MseI were used to digest the products of nested PCR tests using the primer pair SCR16F1A/ScR16R2 [123]. RFLP analysis of the PCR-amplified sequences digested with these restriction enzymes revealed that the sequences obtained from the carrot samples tested positive for *S. citri* have identical restriction profiles to those of the *S. citri* reference strain with all four enzymes and a different profile from that of the *S. citri* reference strain (Cir3B isolated from beet leaf) with all four enzymes [123].

To detect *S. citri*, a droplet digital PCR test (ddPCR) was developed [125]. A comparison of ddPCR and real-time PCR revealed a difference in detection sensitivity [125]. Two sets of spiralin and SpV1 open reading frame 1 (ORF1) primers/probes were also compared [122,125,126]. For the identification of *S. citri* in both culture media and field samples, ddPCR is more accurate than real-time PCR. In a 20 µL reaction, this approach enables the absolute quantification of one copy of the target [125]. It is worth noting that ORF1 primers, which target the SpV1-ORF1 prophage (X51344), are more robust than SP1 primers (targeting the spiralin gene) in CSD diagnosis, according to both real-time PCR and ddPCR data [125].

A loop-mediated isothermal amplification (LAMP) PCR has recently been developed as a highly specific approach for detecting *S. citri*. The target DNA may be detected to a concentration of 100 fg/L using this approach, which is unaffected by crude plant extracts. This LAMP assay, on the other hand, is nine times less sensitive than real-time PCR with pure DNA templates [127]. 

Table 4 provides a comparison of the detection methods described above in terms of both sensitivity and specificity.

## 6. Strategies to Control the Disease

### 6.1. S. citri Sanitation

Some infections, such as *S. citri*, are difficult to remove from the citrus mother plant, according to preliminary research. Their eradication does necessitate the use of specific sanitation techniques [132]. The ability of in vitro shoot-tip grafting to assure the eradication of *S. citri* and the development of healthy citrus stocks has been investigated. It has proven to be a very effective strategy for eradicating citrus graft-transmissible diseases, such as CSD (with a 100% success rate) [133]. The *S. citri* was eradicated from four citrus cultivars using this approach, including Madam Vinous, Navel orange, Valencia orange, and Redblush grapefruit. The results revealed that all plants derived from *S. citri*-infected shoots of the four cultivars and kept for a year under warm circumstances were CSD-free [96].

### 6.2. Cultural Practices

Preventing *S. citri* from achieving and infecting young sensitive plants [50] is the most effective way to avoid CSD, especially in the early-nursery phase [1]. The most efficient way to avoid infection with *S. citri* is to follow a variety of cultural practices [50]. CSD management begins at the nursery, where weeds are monitored, stunted nursery trees are removed, and citrus nursery plants are kept under a screen. All of those precautions are critical to avoid primary infection with *S. citri*. This is because the major source of infection is vectors feeding on weeds and then transferring to young nursery plants when the weeds dry up [1]. Furthermore, because *S. citri* is disseminated by several insect vectors, trap plants appear to be a viable method of limiting CSD propagation. This is the case, for example, with sugar beet, a plant that attracts insect vectors, particularly the beet leafhopper, but is resistant to CSD. Because older trees are less vulnerable to *S. citri* infection, preventing infection while the tree is still growing is critical. As a result, young trees (up to 6 years old) should be removed because they will never produce fruits. However, for infected trees older than six years, an individual assessment should be performed to determine whether symptomatic areas should be removed or the diseased trees should be replaced with healthy trees [50]. CSD can be passed down through grafting. As a result, it is crucial to make sure the mother tree is free of *S. citri* before starting the propagation process. The use of plants obtained from *S. citri*-free areas is essential for avoiding the disease’s introduction into a new orchard. Furthermore, it is critical to keep an eye on weeds in these groves to ensure that they are not disease-prone hosts and to eradicate any susceptible ones as soon as possible [50].

### 6.3. Chemotherapy

In the past, insecticides and tetracycline-based antibiotics were employed to remove insect vectors and alleviate symptoms in spiroplasma-infected plants, respectively [134]. 

The antibiotic sensitivity of *S. citri* has been widely studied, and antibiotics have been utilized to suppress this wall-less pathogen in the field [135]. Indeed, previous research suggests that erythromycin, tylosin, and numerous other antibiotics in the tetracycline group may be effective in the treatment of CSD [136]. The effects of tetracycline compounds, which have an impact on protein synthesis pathways [137], on the development of CSD symptoms were studied in terms of uptake, translocation, and impact. These compounds, applied to the roots as a dip or in hydroponic culture, fully reduced stubborn symptom development in infected seedlings. However, tetracycline compounds in the form of quartz sand drenches were found to be ineffective in preventing CSD symptom development. It is important to highlight that Achromycin, which looked to be more stable than Aureomycin, was more effective in preventing the onset of symptoms [138]. Although several antibiotics have been proven effective in the treatment of CSD *in vitro*, it is important to remember that factors such as antibiotic stability and translocation in plants can have a significant impact on antibiotic performance in vivo when compared to their activity in vitro [136]. Field investigations show that after a lengthy period of use on yellows-diseased plants, including CSD, tetracyclines eventually lose their effectiveness. Antibiotic-resistant *Spiroplasma* strains were suspected to arise in tetracycline-treated diseased plants [135].

The susceptibility of *S. citri* to a variety of systemic insecticides (cygon, furadan, and lannate) and fungicides (benomyl, thiabendazole, and thiophanate M) has been investigated in vitro. *S. citri* was resistant to most of the three systemic insecticides tested. Only thiabendazole in 5% dimethylsulfoxide showed an inhibitory effect equivalent to that of some antibiotics [136].

Although, like other phloem-colonizing and insect-transmitted bacterial pathogens, *S. citri* has shown antibiotic sensitivity in vitro and remission of disease symptoms *in planta*, there is no practical therapy for CSD once a tree is infected [135,136,138]. Furthermore, the widespread use of such biocides in the field is not only ineffective but also environmentally unsound, as antibiotic and insecticide resistance would surely develop in spiroplasmas and their insect vectors [134]. Therefore, to avoid the use of antibiotics in agriculture and the emergence of resistant microbial strains, it is advised that molecules with various modes of action, such as ribosome-inactivating proteins, plant hormones, and resistance inducers such as plasma-activated water, should reasonably be expected [137]. To summarize, it seems that CSD effective control is mainly reliant on preventative and roughing measures, which are themselves reliant on precise and early detection [80].

### 6.4. Genetically Engineered Resistance

There are currently no plants that show natural resistance to *S. citri*. Artificial resistance in plants is now achievable thanks to advances in molecular biology and biotechnology [134]. The *in planta* expression of genes encoding antimicrobial peptides (AMPs), a novel class of antimicrobial compounds that offers an alternative to standard antibiotics, is one of the ways to engineer cellular pathogen resistance [139]. In other words, employing AMPs to engineer artificial plant resistance has been viewed as a possible approach for controlling agronomically significant spiroplasmal diseases, including CSD. A recent study focused on screening AMPs that have the potential to inhibit the growth of *S. citri*. For the in vitro growth inhibition test, four AMPs were selected: Novispirin T7, Caerin 1.1, Tricholongin, and Dhvar4. For rapid qualitative and quantitative analyses of the AMPs, a liquid assay method was designed. Novispirin T7 and Caerin 1.1 inhibited the growth of *S. citri* with efficacy comparable to tetracycline. Spiroplasma cultures treated with these two peptides showed cell deformations, indicating that the AMPs interact with the spiroplasma cell membranes. Because Novispirin T7 and Caerin 1.1 are both short, linear peptides that are water-soluble, they can be synthesized chemically and supplied exogenously. Alternatively, using a gene expression cassette, the peptides can be engineered into spiroplasma-susceptible plants. The expression of engineered AMPs in plants may improve plant resistance against CSD [134]. 

## 7. Disease Situation in the Mediterranean Region: Focus on Morocco

Citrus stubborn is a common disease in the Mediterranean. *S. citri* has been found in nearly every Mediterranean country and is one of the most common citrus infections [140]. Furthermore, in the Mediterranean region, citrus stubborn is considered an endemic vector-borne disease [2]. The development of reliable diagnostic procedures has enabled extensive surveys of *S. citri* in various areas of the region. *S. citri* was identified in several countries, including Morocco [5,14,43,69,114,141,142,143,144], Algeria [43,145], Tunisia [43,146,147,148], Egypt [44], Syria [43,49,69], Lebanon [43], Palestine [69], Israel [4,43], Turkey [4,43,45], Spain [4], Italy [4,57], Cyprus [63], and France [149]. In Italy and Spain, CSD appears to be of minor importance [2], while some citrus-growing Mediterranean countries, such as Malta, Croatia, and Portugal, have been reported to be free of *S. citri* [150]. The natural transmission of *S. citri* by different leafhopper species and the deployment of buds from infected trees are thought to be the key explanatory causes underlying the prevalence of CSD in the Mediterranean area [69]. 

In Morocco, stubborn was once thought to be one of the rare citrus diseases [142]. The disease’s causal agent has been found in every citrus-growing region of the country, including Gharb, Haouz, Loukkos, Moulouya, Souss [143], and Tadla [5,141,143]. In Morocco, a genetic investigation of *S. citri* has never been performed. However, it should be underlined that a first complete nucleotide sequence of the circular chromosome, as well as two plasmids from *S. citri* of Moroccan origin, have been recently documented [14].

## 8. Conclusions and Future Prospects

Although CSD has been studied for decades, the accurate detection of the disease remains difficult due to the disease’s uneven distribution and low titers in diseased trees, as well as considerable seasonal fluctuations [103,122,151]. In other words, a variety of factors, such as sampling season and growing circumstances, as well as *S. citri* strains, can influence the accuracy of disease diagnosis [124]. Even though biological indexing is required in certification programs, the limitations of traditional biological indexing, such as the low concentration of *S. citri*, the pathogen’s unsatisfactory transmission rates in the greenhouse, and the long delay in symptom expression, have limited its widespread use [46]. It is worth noting that the use of ScCCPP1 is the first documented serological diagnosis of CSD that is not dependent on the presence of *S. citri* in the analyzed sample [80]. Briefly, it is not easy to choose one CSD diagnosis approach over another, even if the results are consistent. The best option will be determined by the diagnosis goals and the facilities of each laboratory.

The control of CSD is still an enigma. This is owing to the airborne transmission of the disease by leafhopper vectors that primarily feed on weeds. Since the discovery that a mollicute is the cause of CSD, significant control attempts have been made. This was accomplished through the use of a variety of methods, including whitewash sprays and preventative net covering. None of these techniques, however, has been demonstrated to be successful in preventing the spread of CSD. This is related to the difficulties of conducting studies with a disease that has a sporadic natural infection [2]. The key to preventing CSD propagation is to stay on top of the sanitary state of citrus donor plants to produce *S. citri*-free propagating material. In vitro shoot-tip grafting is successful in removing *S. citri* from propagating material [96,132,133].

In terms of the current situation of *S. citri* across Morocco, this paper presents an overview of the disease’s spread in the citrus-growing regions of the country. Virus-free (certified) saplings, vector management, regular monitoring of citrus orchards to enable the early diagnosis of the disease, and grubbing up of affected trees could all help to prevent the introduction and spread of CSD in Moroccan citrus groves. This overview includes references to many disciplines of research on CSD in Morocco and worldwide. 

The characterization of *S. citri* strains, the identification of potential leafhopper vectors, the search for secondary hosts, and the development of sustainable control measures are some of the research topics that could be pursued as prospects. Investigating functional genomics in the citrus–*S. citri* interaction using transcriptomic and/or proteomic approaches would be an interesting way to learn more about the full mechanisms underlying the complex and varied events associated with such an interactome and thus aid in the development of new diagnostic methods and plant protection strategies. The relationship between *S. citri* and other citrus pathogens (viruses and viroids) prevailing in Moroccan citrus orchards [152,153,154] should be deeply investigated in the future to learn more about the whole range of interactions that these pathogens have during infection and their impact on CSD severity. This advanced research will contribute to a better knowledge of the epidemiology of *S. citri* and the mechanisms behind its spread worldwide, including Morocco, therefore helping to develop novel pathogen control measures.

## Figures and Tables

**Table 1:** Results summary of the known field trials carried out in different citrus-growing countries to evaluate the effect of *Spiroplasma citri* on vegetative growth and yield of different citrus scion and rootstock combinations.

Country (Region)	Study Period	Combination		*S. citri* Infection	Effect on				References
		Scion	Rootstock		Height	Canopy	Fruit Drop	Yield	
United States (Central California)	2006–2007	Navel sweet orange	Carrizo citrange (*Citrus sinensis* Osb. × *Poncirus trifoliata* L. Raf.)	Naturally infected by leafhoppers	SE (reduction of almost 27% in 2007).	SE (reduction of almost 12% in 2007).	SE (3 and 4.7 fold higher than *S. citri*-free trees in 2006 and 2007, respectively).	SE (reduction of almost 52 and 45% in 2006 and 2007, respectively).	[52]
United States (California)	DN	Navel sweet orange	-	Graft inoculation	SE (reduction of almost 55%).	-	-	-	[64]
United States (Central California)	1982–1983	Navel sweet orange	Rough lemon	Naturally infected	-	-	-	SE (diseased trees produce an average of 20 Kg less than the healthy ones).	[62]
Cyprus (Akhelia)	1984–1994	Frost Washington Navel sweet orange	Sour orange	Naturally infected by leafhoppers	NSE	-	-	SE (reduction of almost 19%).	[63]
		Frost Valencia sweet orange	Sour orange	Naturally infected by leafhoppers	SE (reduction of almost 11%).	-	-	SE (reduction of almost 34%).	[63]
Italy (Sicily)	DN	Eureka lemon	Sour orange	Graft-inoculation	Growth was retarded.	-	-	-	[57]

SE: Significant effect. NSE: No significant effect. DN: Data not shown.

**Table 3:** Primer sequences and their annealing temperature (Tm), primer/probe location, and expected size of PCR products for each primer pair when used to amplify *Spiroplasma citri* by PCR and related tests.

	Sequence	Tm	Targeted Gene/ Region	Genomic Coordinates	Size of the Expected Product	References
**PCR**						
Spiralin-f	5′-GTCGGAACAACATCAGTGGT-3′	60 °C	Spiralin	55–74	675 bp	[122]
Spiralin-r	5′- TGCTTTTGGTGGTGCTAATG- 3′			710–729		
P58-6f	5′- GCGGACAAATTAAGTAATAAAAGAGC-3′	56 °C	Putative P58 adhesin-like	445–470	450 bp	
P58-4r	5′-GCACAGCATTTGCCAACTACA-3′			874–894		
P89-f	5′-ATTGACTCAACAAACGGGATA- 3′	56 °C	Putative P89 adhesion	5786–5807	707 bp	
P89-r	5′-ACGGCGTTTGTTAATTTTTGGTA 3′			6471–6492		
D	5′-GTATAAAGTAGGGTTAGAAGC-3′	57 °C	Spiralin	-	1053 bp	[120]
D’	5′-CCCTTGTGAATCACCACC-3′					
Scif	5′-AACAACTCAATTATCACTTTG-3′	54 °C	pE gene of pSci1 plasmid	-	422 bp	[124]
Scif	5′-AACAACTCAATTATCACTTTG-3′					
**Nested PCR**						
ScR16F1/	5′-AGGATGAA CGCTGGCGGCAT-3′	50 °C	16S rDNA	-	1500 bp	[123]
ScR16R1	5′-GTAGTCACGT CCTTCATCGT-3′					
ScR16F1A/	5′- GCATGCCTAATACATGCAAG-3′					
ScR16R2	5′-ATC CATCCGCACGTTCTCGTAC-3′					
**Real-time PCR**						
P58-3f	5′-GTCCCTAATGCACCGTGAAAA-3′	56 °C	Putative P58 adhesin-like	776–796	119 bp	[122]
P58-4r	5′-GCACAGCATTTGCCAACTACA-3′					
SP1 F (209–232)	5′-AAGCAGTGCAAGGAGTTGTAAAAA-3′	54 °C	Spiralin	209–288	79 bp	[122]
SP1 R (261–288)	5′-TGATGTACCTTTGTTGTCTTGATAAACA-3′					
SP1 P (242–259)	5′-6FAM/CAGCTGATTTTCAATTTG/MGB/NFQ-3′					[125]
ORF1F (777–798)	5′-TGGCAGTTTTGTTTAGTCATCC-3′	57/58 °C	SpV1-ORF1 Prophage	777–966	190 bp	[126]
ORF1R (946–966)	5′-GGGTCTAAACGCCGTTAAAGT-3′					
ORF1P	5′-6FAM/TTGGGTTTGGTTATTCCATT/MGB/NFQ-3′					[125]
CCPPscitriJF D-F	5′-ATTGCAGCACCTGC AACTGTAG-3′	-	Spiralin	-	-	[80]
CCPPscitriJFD-R	5′-TGTTTTTACAA CTCCTTGCACTGC-3′					
CCPPscitriJFD-P	5′-FAM -AC AGCGTTAGAAGCTAAT-3′					
**LAMP PCR**						
F3	5′-ACAGCAAACCCAAAACAAG-3′	47 °C	Spiralin	-	-	[127]
B3	5′-CAACAGTTTTATCTTTTGCTGGAG-3′	52 °C		-	-	
FIP	5′-CTGCTGTTGCTGTTTTTACAACTCTTTTGCTGAAATTAAAACAGCGTTAGAAGC-3′	68 °C		-	-	
BIP	5′-CAATTTGATGTTTATCAAGACAACTTTTACTTCAACGTTACCTCCTT-3′	65 °C		-	-	
LB	5′-LB GGTRMATCATTAACAACAAT-3′	39 °C		-	-	

**Table 4:** Summary of the main findings from the different *Spiroplasma citri* detection tests.

**Tested Methods** **Main Findings**	**References**
**Isolation of *S. citri* in Culture Medium**	
**C-3G** ▪ The presence of *S. citri* in the tested sample is indicated by a change in the color of the infected liquid media from red to yellow. ▪ The appearance of typical fried-egg shape colonies on C-3G medium containing 0.8% of agar and fuzzy colonies with occasional surrounding satellite colonies due to the ability of spiroplasma cells to move through the agar matrix.	[5,44,129]
**SP4** ▪ Isolation attempts from leafhoppers, plants, and flowers. ▪ A complex medium used for primary and maintenance of *S.citri*. ▪ Growth medium SP4 gives an isolation rate of 100% from continuous egg-passaged lines. ▪ Growth of *S. citri* is more rapid and has a higher titer with 5% or 10% of horse serum than 20%.	[106,107]
**R2** ▪ Spiroplasmas can grow in simplified media that contain only PPLO broth base, horse serum, and carbohydrates. ▪ Cell morphology in log phase growth in R2 and C-3G was comparable to that in M1D. ▪ Differences in the growth curves of seven spiroplasmas appeared to be more closely related to species identity than to the media in which they were grown. ▪ R2 is a simplified media especially appropriate for high volume procedures, such as liquid dilution cloning and antigen pellet production for antisera. ▪ R2 sustained spiroplasma growth consistently through 10 subcultures (100% of cultures survived) with good helical morphology.	[5,105,106,130]
**LD8** ▪ Highly suitable for the primary isolation of *S. citri* in in vitro and from infected plants. ▪ The maximum titer of *S. citri* grown in LD8 medium 6 × 10^9^ colony-forming unit CFU/mL with an estimated doubling time of about 4 h.	[105]
**ELISA**	
▪ Several symptomatic field samples were ELISA negative, probably due to the low sensitivity of the method to detect low titers of the pathogen or because of the uneven distribution of the pathogen in the plant. ▪ ELISA protocol can provide inconsistent results when using citrus leaves as samples. ▪ Neither culture tests nor ELISA permitted the detection of *S. citri* in asymptomatic citrus plants. ▪ Failure to detect the agent in the early stages of the plant disease is a result of the low number of spiroplasmas present in the phloem.	[118,124]
**Conventional PCR**	
▪ The sensitivity of this detection method was 100 to 1000 times higher than that of ELISA or culture assay. ▪ Diagnosis using primers designed from the P89 or P58 genes is 1000 times more sensitive than that with the spiralin gene. ▪ Spiralin primer pair gives good results in the hottest period of the summer (August). ▪ Assay sensitivity was estimated to be 8 × 10^−5^ to 1.2 × 10^−6^ ng of *S. citri* DNA (6.14 × 10^5^ to 9.6 × 10^3^ copies of target gene) per milligram of tissue collected from field citrus trees. ▪ *S. citri* titer was consistently higher in fruit columella than in leaf midribs, making the former tissue the best choice for sampling. ▪ PCR detected *S. citri* from culture-negative trees in 5 to 15% of cases. ▪ Scif⁄Scir primer pair for *S. citri* detection based on pE gene of pSci1 plasmid was more sensitive than that based on spiralin gene or 16S rDNA. ▪ Inoculum collection for transmission tests and sampling to detect *S. citri* can be limited to the hot summer months when *S. citri* titer is generally highest.	[44,46,51,118,120,122,124,128,131]
**Nested PCR**	
▪ Nested PCR of the spiralin gene based on primer pairs D⁄D’ followed by F1⁄R1 detects 36.6% infection in symptomatic samples.	[120,128]
**Real-time PCR**	
▪ The best tissue for the detection of *S. citri* is fruit columella because the pathogen titer was highest in this tissue. ▪ The result of the real-time PCR test was significantly correlated to disease status (mildly or severely symptomatic). ▪ The sensitivity of the primer pair P58-3f/P58-4r is 8 × 10^−5^ to 1.2 × 10^−6^ ng of *S. citri* DNA/mg of tissue. ▪ The efficiency of the real-time PCR assay was about 95.2% (R2 = 0.999). ▪ Php-orf1 (primers of prophage sequences) improve sensitivity by 4.91 and 3.65 cycle threshold (Cq) units compared with housekeeping gene primers for spiralin and P58 putative adhesin genes.	[120,122,123,126,128,131]
**IC-PCR**	
▪ Simplifies sample preparation and enhances the specificity and sensitivity of conventional PCR. ▪ More sensitive technique to detect spiroplasmas than ELISA and cultivation. ▪ A sensitive and specific technique. ▪ Problems of sample contamination. Therefore, extreme care must be taken to avoid false-positive reactions.	[118,121]
**RFLP**	
▪ With all four enzymes utilized, RFLP analysis, amplified by nested PCR with *S. citri* 16S rDNA-based primers, showed that carrot samples that tested positive for *S. citri* exhibited identical restriction profiles to those of the *S. citri* reference strain.	[123]
**LAMP**	
▪ Targeting the spiralin gene. ▪ Sodium acetate (NaOAc) buffer 50 mM was selected as best for crude extract preparation. ▪ The limit of detection of the LAMP assay was 100 fg/μL for the pure plasmid DNA and 100 fg/μL for the pure DNA incorporated in healthy plant extract but was approximately 9-times less sensitive than the standard real-time PCR technique targeting the spiralin gene. ▪ A simplified procedure using crude extracts applied directly for LAMP analysis allows on-site diagnostic capability that can largely overcome limitations for large-scale screening. ▪ In comparison to real-time PCR, LAMP is at least 10-times faster and can be used in both the laboratory and field. ▪ The LAMP assay showed high specificity to *S. citri* and detected DNA to a level of 100 fg/μL with no inhibition by crude plant extracts.	[127]
**Biological Indexing**	
▪ Biological indexing for *S. citri* involves graft inoculation of tissue into sensitive varieties, such as Madam Vinous sweet orange. ▪ Symptoms of CSD were obtained only under warm conditions. ▪ Constraints of traditional biological indexing are associated with the low concentration of *S. citri*, the unsatisfactory transmission rates of the pathogen in the greenhouse, and the long delay in the onset of symptoms. ▪ The “inverse inoculation” is more efficient than the traditional inoculation method.	[46,59,96]

## Data Availability

The data used for the analyses in this study are available within the article.
